# A propensity score matching study: The prevalence of mental health problems among pregnant women at first antenatal care increased in Chongqing during the first wave of the COVID-19 pandemic

**DOI:** 10.3389/fpubh.2023.1142461

**Published:** 2023-04-14

**Authors:** Jiamei Guo, Xiao Li, Jinglan He, Ming Ai, Yao Gan, Qi Zhang, Anhai Zheng, Wanjun Chen, Lulu Chen, Sisi Liang, Xiaoyu Yu, Li Kuang

**Affiliations:** ^1^Department of Psychiatry, The First Affiliated Hospital of Chongqing Medical University, Chongqing, China; ^2^Department of Obstetrics, The First Affiliated Hospital of Chongqing Medical University, Chongqing, China

**Keywords:** prevalence, mental health problems, COVID-19, propensity score matching, pregnant women

## Abstract

**Background:**

The 2019 coronavirus disease (COVID-19) pandemic increased the risks of mental health challenges, especially anxiety and depression. However, the impact of COVID-19 on mental health during pregnancy has not been fully established. Therefore, we investigated the impact of the COVID-19 pandemic on maternal mental health.

**Methods:**

Two cohorts of pregnant women at their first antenatal care in the First Affiliated Hospital of Chongqing Medical University were enrolled in this study. One cohort was enrolled before the COVID-19 outbreak, from 1 June to 31 December 2019 (*n* = 5,728, pre-COVID-19 group), while the other was enrolled during the COVID-19 pandemic, from 24 January to 23 March 2020 (*n* = 739, COVID-19 pandemic group). Symptoms of depression, anxiety, and somatization disorders were assessed by the Patient Health Questionnaire-9 (PHQ-9), Generalized Anxiety Disorder-7 (GAD-7), and Patient Health Questionnaire-15 (PHQ-15), with a cutoff point of 10 for moderate-to-severe depression, anxiety, and somatoform symptoms. The propensity score matching method (1:1) was used to balance differences in demographic characteristics between groups. A chi-square analysis was performed to compare differences in demographic characteristics between the groups.

**Results:**

Prevalence of moderate-to-severe depression, anxiety, and somatoform symptoms among pregnant women at their first antenatal care visit during the COVID-19 pandemic (9.5, 2.2, and 20.8%, respectively) was significantly lower than those before the pandemic (16.3, 4.4, and 25.7%, respectively) (*p* < 0.05). Compared with the same period before the pandemic, during the pandemic, the number of women newly registered for antenatal care decreased by nearly 50%. There were significant differences in the distributions of demographic characteristics between the groups (*p* < 0.05). After matching the demographic characteristics, differences in the prevalence of maternal mental health disorders between the groups reversed dramatically. Prevalence of moderate-to-severe depression, anxiety, and somatoform symptoms during the COVID-19 pandemic in this population (2.3, 9.6, and 20.8%, respectively) was significantly higher than those before the pandemic (0.3, 3.9, and 10%, respectively) (*p* < 0.05).

**Conclusion:**

The COVID-19 pandemic increased mental health risks among pregnant women. As a large proportion of pregnant women with mental health challenges delay their prenatal care or change healthcare facilities after the outbreak of public health emergencies, there is a need to establish a balanced healthcare system in medical institutions at all levels.

## Introduction

The COVID-19 pandemic presents a significant threat to the physical and mental health of patients, especially anxiety and depression (1, 2). Previously, we found that 20.7 and 30.2% of hospital workers experienced anxiety and depression during the COVID-19 pandemic (3). Yang et al. reported that the prevalence of anxiety and depression in the general population with different sociodemographic backgrounds was 12.6 and 24.3%, respectively (4). Moreover, during the pandemic, students exhibited relatively high incidences of anxiety and depression (5–7). Wang et al. revealed that 53.8% of their study participants ranked the psychological impact of COVID-19 from moderate to severe (8). Therefore, the impact of life-threatening public health emergencies on mental health is enormous and extensive.

Pregnancy is a normal physiological state in women of childbearing age. Due to hormonal changes, role transitions, and other psychosocial factors, pregnant women are more likely to develop depression and anxiety than non-pregnant women (9). Almost 16.3% of pregnant women experience depression during pregnancy, and the figure may be higher in low-and middle-income countries (10, 11). Untreated antenatal depression and anxiety may result in a series of short-and long-term negative effects on mothers, infants, and their families (12–14). Pregnant women are at higher risk for severe COVID-19 infections, which may increase the likelihood of preterm births (15–17). Moreover, during the COVID-19 pandemic, pregnant women exhibited higher risks for anxiety and depression than non-pregnant women (18). Mo et al. reported that the prevalence of depression and anxiety in pregnant women was 48.7 and 33.0%, respectively, and more than two-thirds of pregnant women showed concerns about COVID-19 (19). Wu et al. found that depression incidences in pregnant women increased after the COVID-19 outbreak, rising from 26 to 34.2%, accompanied by a significant increase in anxiety symptoms (20). Zhou et al. reported inconsistent findings, showing that pregnant women had fewer anxiety and depression symptoms and were less worried about being infected with COVID-19, compared to non-pregnant women (21, 22). Overall, evidence supports that the COVID-19 pandemic exerted severe negative impacts on the mental health of pregnant women (23–25). Therefore, timely screening and counseling interventions for depression and anxiety among pregnant women are crucial during the COVID-19 pandemic.

Most of the current studies on maternal mental health during the COVID-19 pandemic focused on anxiety and depression among pregnant women and their associated risk factors (26–28). To the best of our knowledge, only five studies compared maternal mental health disorders before and after the COVID-19 outbreak [two from China (20, 29), one from Saudi Arabia (30), one from Canada (31), and one from Turkey (32)]. Among them, two studies compared differences in the prevalence of mental health disorders in the same group of pregnant women at two time points (pre-and post-COVID-19 outbreak), with small sample sizes (101 and 63 cases), while the remaining three studies compared different groups of pregnant women recruited at two time periods (pre-and post-COVID-19 outbreak). Given the limited number of studies, there is a need to elucidate depression and anxiety incidences among pregnant women during the COVID-19 pandemic. In May 2019, our hospital established an obstetrics multi-disciplinary team clinic that consisted of psychiatrists, obstetricians, and psychological consultants and conducted a free online mental health assessment for all pregnant women at their first antenatal care visit to our obstetrics clinic. The COVID-19 outbreak in 2020 provided us with coincidental data on maternal mental health pre-and post-COVID-19 outbreak, which made it possible to explore the impact of the COVID-19 pandemic on maternal mental health from a real-world perspective. Our findings provide valuable information and a basis for relevant policy-making after public health emergencies.

## Methods

### Study design and population

This was a cross-sectional study performed in Chongqing, southwest China. Study participants were enrolled from the department of obstetrics, the First Affiliated Hospital of Chongqing Medical University, from 1 June to 31 December 2019 (pre-COVID-19 period) and from 24 January to 23 March 2020 (COVID-19 pandemic period). Pregnancy was established by B-ultrasonography, and the women were required to complete the free online psychological assessment questionnaire at their first prenatal care visit. Pregnant women who did not partake in the assessment or who were illiterate were excluded from this study. Written informed consent was obtained from all participants before evaluation. This study was approved by the ethical committee of Chongqing Medical University, China.

### Data collection and quality control

Data collection and quality control were performed as previously described (33). General information and psychological assessment data for pregnant women were obtained through the exclusive QR code and website. Access to online assessments was restricted as the unique telephone number for each pregnant woman was used for the log-ins. The online questionnaire comprehensively introduced the aim of the project and provided the assessment instructions for each specific scale. All participants completed these assessments on their own, with two trained nurses on hand to answer any questions they had. After submission of the assessments, the nurse wrote down the scale scores.

All entries were set as compulsory questions, and the IP address verified by the mobile phone of the tester could only save the final answer on the test day. Questionnaires would only be submitted after completing all items. Otherwise, the system would automatically identify the outcome as incomplete. Online psychological assessments have been adopted for depression screening among pregnant women (33). We set the test time based on the pre-test results and deleted the questionnaires whose test duration was <180 s.

### Demographic information

Participants’ basic demographic information, including age, last menstrual period, residence (rural vs. urban), nationality (Han vs. minority), marital status (married, unmarried, or divorced), level of education (middle school or lower, high school, college, and master’s degree or higher), occupation (fixed or self-employed and unemployed), parity (nulliparous vs. multiparous), and gestational weeks, was collected at interview.

### Assessment of depression, anxiety, and somatization symptoms

The Chinese version of Patient Health Questionnaire-9 (PHQ-9) is a 9-item scale that is used to evaluate depressive symptoms in the preceding 2 weeks before assessment, with scores ranging from 0 to 3 for each item: 0 = “none,” 1 = “several days,” 2 = “more than half the days,” and 3 = “nearly every day.” Findings were categorized as follows: 0–4 as normal, 5–9 as mild depression, 10–14 as moderate depression, 15–19 as moderate-to-severe depression, and 20–27 as severe depression. A cutoff total score of 10 was defined as depression, while sensitivity and specificity were 88 and 86%, respectively (34). In this study, Cronbach’s alpha for PHQ-9 was 0.87.

The Chinese version of Generalized Anxiety Disorder-7 (GAD-7) was used to evaluate the severity of anxiety in the preceding 2 weeks. GAD-7 uses a four-point Likert scale (35) with scores ranging from 0 to 3 for each item: 0 = “none,” 1 = “several days,” 2 = “more than half the days” and 3 = “nearly every day.” Findings were categorized as follows: 0–4 as normal, 5–9 as mild anxiety, 10–14 as moderate anxiety, and 15–21 as severe anxiety. The positive screening for anxiety symptoms was defined with a cutoff score of 10 or higher, sensitivity of 89%, and specificity of 82% (36). In this study, Cronbach’s alpha for PHQ-9 was 0.84.

The Chinese version of Patient Health Questionnaire-15 (PHQ-15) was used to assess somatic symptoms in the preceding 4 weeks, with scores ranging from 0 to 2 for each item: 0 = “no disturbance,” 1 = “little disturbance,” and 2 = “much disturbance” (37). Findings were categorized as follows: 0–4 as normal, 5–9 as mild somatic symptoms, 10–14 as moderate somatic symptoms, and 15–30 as severe somatic symptoms. A cutoff point of ≥10 was used to assess the presence of somatic symptoms, with a sensitivity of 80.2% and a specificity of 58.5% (38). In this study, Cronbach’s alpha for PHQ-9 was 0.86.

### Statistical analysis

Data were analyzed using the IBM SPSS 22.0 software. Descriptive statistics were used to present the study variables and demographic characteristics. A chi-square analysis was used to compare differences between groups. The propensity score matching method (1:1) was used to balance differences in distributions of demographic characteristics, with the grouping variable as the dependent variable (COVID-19 pandemic = 1, pre-COVID-19 = 0), demographic characteristics (age, residence, marital status, education, occupation, pregnancy weeks and gravidity) as covariates, and matching tolerance as 0 for categorical variables. A total of 710 accurate matching pairs were obtained for analyses. A *p*-value ≤ 0.05 was the threshold for significance.

## Results

Between 1 June and 31 December 2019 (pre-COVID-19), 5,780 pregnant women completed the online psychological assessment at their first antenatal care visit. Of those, 52 women were excluded due to being in the postpartum period or due to missing any results from PHQ-9, GAD-7, or PHQ-15. Thus, 5,728 questionnaires were analyzed. Between 24 January and 23 March 2020 (during the COVID-19 pandemic), 747 pregnant women finished the online questionnaire. Eight questionnaires were excluded either because the women were in the postpartum period or because of missing results from any scale. A total of 739 questionnaires were analyzed. Most of the enrolled participants were aged between 25 and 34 years (*n* = 4,764; 73.7%), were of Han nationality (*n* = 6,119; 94.6%), were married (*n* = 5,745; 88.8%), were employed (*n* = 5,306; 82%), and were in their first trimesters (*n* = 4,672; 72.2%) (Table 1).

**Table 1:** Demographic characteristics of the two groups.

Characteristic	Total sample (*n* = 6,467)	COVID-19 pandemic (*n* = 739)	Pre-COVID-19 (*n* = 5,728)	*χ*^2^/*F*	Value of *p*
Age, No. (%)				21.704	<0.001*
≤24	729 (11.3)	75 (10.1)	654 (11.4)		
25–29	2,638 (40.8)	316 (42.8)	2,322 (40.5)		
30–34	2,126 (32.9)	258 (34.9)	1,868 (32.6)		
35–39	653 (10.1)	78 (10.6)	575 (10.0)		
≥40	321 (5.0)	12 (1.6)	309 (5.4)		
Residence, No. (%)				2.562	0.109
Rural	2,935 (45.4)	315 (42.6)	2,620 (45.7)		
Urban	3,532 (54.6)	424 (57.4)	3,108 (54.3)		
Race, No. (%)				4.891	0.027*
Han nationality	6,119 (94.6)	712 (96.3)	5,407 (94.4)		
Others	348 (5.4)	27 (3.7)	321 (5.6)		
Marital status, No. (%)				18.34	<0.001*
Married	5,745 (88.8)	691 (93.5)	5,054 (88.2)		
Unmarried/divorce	722 (11.2)	48 (6.5)	674 (11.8)		
Education, No. (%)				31.975	<0.001*
Middle school or less	622 (9.6)	52 (7.0)	570 (10.0)		
High school	921 (14.2)	103 (13.9)	818 (14.3)		
Technical secondary school	2,064 (31.9)	294 (39.8)	1,770 (30.9)		
College	2,400 (37.1)	229 (31)	2,171 (37.9)		
Master or higher	460 (7.1)	61 (8.3)	399 (7.0)		
Gravidity, No. (%)				10.127	0.001*
Primigravida	2,806 (43.4)	361 (48.8)	2,445 (42.7)		
Multigravida	3,661 (56.6)	378 (51.2)	3,283 (57.3)		
Occupation, No. (%)				51.801	<0.001*
Fixed/self employed	5,306 (82)	677 (91.6)	4,629 (80.8)		
Not employed	1,161 (18)	62 (8.4)	1,099 (19.2)		
Gestational weeks, No. (%)				60.433	<0.001*
First trimester (<14)	4,672 (72.2)	493 (66.7)	4,179 (73.0)		
Second trimester (14–28)	1,249 (19.3)	214 (29)	1,035 (18.0)		
Third trimester (>28)	546 (8.4)	32 (4.3)	514 (9.0)		

*p*-values were calculated using the chi-square test.

* Statistically significant: *p* < 0.05.

During the COVID-19 pandemic, 272/739 (36.8%) pregnant women exhibited different degrees of depressive symptoms. Prevalence of mild, moderate, moderate-to-severe, and severe depressive symptoms was 27.3, 7.6, 1.6, and 0.3%, respectively. A total of 111/739 (15%) pregnant women had different degrees of anxiety symptoms. The prevalence of mild, moderate, and severe anxiety symptoms was 12.9, 1.8, and 0.4%, respectively. The prevalence of moderate-to-severe depression (PHQ-9 ≥ 10), anxiety (GAD-7 ≥ 10), and somatoform symptoms (PHQ-15 ≥ 10) during the COVID-19 pandemic was 9.5% (*n* = 70), 2.2% (*n* = 16), and 20.6% (*n* = 152), respectively. Before the COVID-19 outbreak, the prevalence of moderate-to-severe depression, anxiety, and somatoform symptoms was 16.3, 4.4, and 25.7%, respectively (Table 2). The prevalence of depression, anxiety, and somatoform symptoms among pregnant women during their first antenatal care during the COVID-19 pandemic was significantly lower than those before the COVID-19 outbreak (*p* < 0.05; Table 2).

**Table 2:** Results of PHQ-9, GAD, and PHQ-15 between the two groups.

Mental health problems	COVID-19 pandemic (*n* = 739)	Pre-COVID-19 (*n* = 5,728)	*χ* ^2^	Value of *p*
PHQ-9 level, No. (%)			52.897	<0.001*
No depression symptoms	467 (63.2)	2,842 (49.6)		
Mild depression	202 (27.3)	1,953 (34.1)		
Moderate depression	56 (7.6)	699 (12.2)		
Moderate–severe depression	12 (1.6)	187 (3.3)		
Severe depression	2 (0.3)	47 (0.8)		
GAD-7 level, No. (%)			27.12	<0.001*
No anxiety symptoms	628 (85.0)	4,396 (76.7)		
Mild anxiety	95 (12.9)	1,080 (18.9)		
Moderate anxiety	13 (1.8)	175 (3.1)		
Severe anxiety	3 (0.4)	77 (1.3)		
PHQ-15 level, No. (%)			19.325	<0.001*
No somatic symptoms	298 (40.3)	1,905 (33.3)		
Mild somatic symptoms	289 (39.1)	2,352 (41.1)		
Moderate somatic symptoms	129 (17.5)	1,165 (20.3)		
Severe somatic symptoms	23 (3.1)	306 (5.3)		
Depression positive			23.207	<0.001*
PHQ-9 ≥ 10	70 (9.5)	933 (16.3)		
PHQ-9 < 10	669 (90.5)	4,795 (83.7)		
GAD positive			8.226	0.004*
GAD-7 ≥ 10	16 (2.2)	252 (4.4)		
GAD-7 < 10	723 (97.8)	5,476 (95.6)		
Somatoform disorders positive			9.101	0.003*
PHQ-15 ≥ 10	152 (20.6)	1,471 (25.7)		
PHQ-15 < 10	587 (79.4)	4,257 (74.3)		

*p*-values were calculated using the chi-square test. PHQ, Patient Health Questionnaire-15; GAD-7, Generalized Anxiety Disorder.

* Statistically significant: *p* < 0.05.

**Figure 1:**
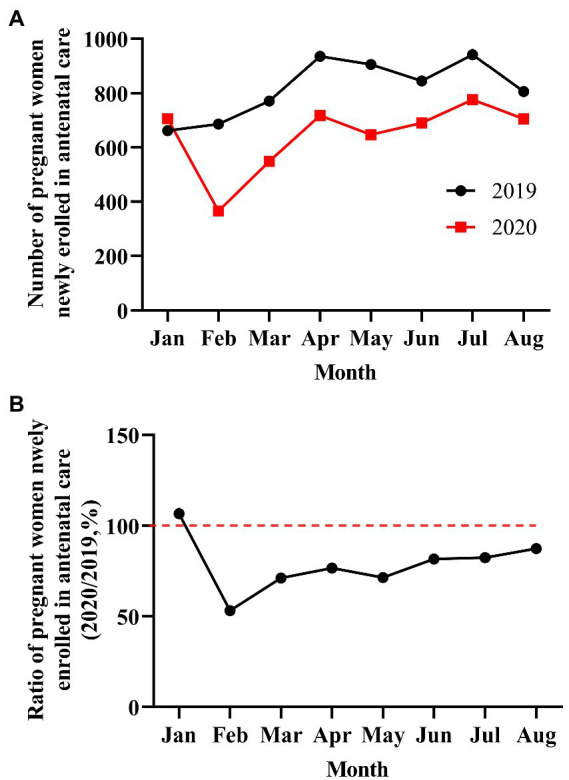
Number and ratio of pregnant women newly enrolled in antenatal care from January to August 2019 and 2020 (pre-and post-COVID-19 outbreak). **(A)** The number of pregnant women from January to August 2019 and 2020. **(B)** The ratio of pregnant women from January to August 2019 and 2020 (2020/2019, %).

To clarify the impact of the COVID-19 pandemic on maternal mental health, we first queried the number of pregnant women who had newly registered for antenatal care in our hospital before, during, and after the COVID-19 pandemic. In Figure 1A and 1B, during the COVID-19 pandemic, the number of pregnant women decreased by nearly 50%, compared with the same period before the pandemic. The number of pregnant women gradually increased over time as the lockdown ended, but it was still down by at least 12% when compared with the same month in the previous year at a timepoint of nearly 6 months after the COVID-19 pandemic. Second, we compared differences in demographic characteristics among pregnant women in the two groups (pre-COVID-19 vs. COVID-19 pandemic). Table 1 shows that there were significant differences in distributions of demographic characteristics (age, Han nationality, education level, gestational weeks, marital status, occupation, and gravidity) among pregnant women in the two groups (*p* < 0.05). Compared to the pre-COVID-19 period, the proportions of pregnant women in the elderly age (>40 years) (1.6% vs. 5.4%), middle school degree or below (7% vs. 10%), ethnic minorities (3.7% vs. 5.6%), multigravida (51.2% vs. 57.3%), unemployed (8.4% vs. 19.2%), and unmarried/divorced (6.5% vs. 11.8%) were significantly lower during the COVID-19 pandemic (*p* < 0.05).

Hence, findings from the two groups with demographic mismatch do not inform on whether the COVID-19 pandemic exerted positive or negative effects on the mental health of pregnant women. To eliminate the effects of demographic distribution differences on analysis, 710 exact matches were obtained *via* propensity score matching (Table 3). There were no significant differences in distributions of age, residence, ethnicity, education level, gravidity, occupation, marital status, and gestational weeks between the two newly matched groups (*p* > 0.05; Table 3). Prevalence of moderate-to-severe anxiety (GAD-7 ≥ 10), depression (PHQ-9 ≥ 10), and somatoform symptoms (PHQ-15 ≥ 10) among pregnant women during the COVID-19 pandemic was 2.3, 9.6, and 20.8%, respectively, significantly higher than those in the pre-COVID-19 period (0.3, 3.9, and 10%) (*p* < 0.05; Table 4).

**Table 3:** Demographic characteristics between the two matched groups.

Characteristic	COVID-19 pandemic (*n* = 710)	Pre-COVID-19 (*n* = 710)	*χ*^2^/*F*	Value of *p*
Age, No. (%)			0	1
≤24	69 (9.7)	69 (9.7)		
25–29	314 (44.2)	314 (44.2)		
30–34	244 (34.4)	244 (34.4)		
35–39	71 (10)	71 (10)		
≥40	12 (1.7)	12 (1.7)		
Residence, No. (%)			0	1
Rural	307 (43.2)	307 (43.2)		
Urban	403 (56.8)	403 (56.8)		
Race, No. (%)			2.01	0.156
Han nationality	684 (96.3)	673 (94.8)		
Others	26 (3.7)	37 (5.2)		
Marital status, No. (%)			0	1
Married	669 (94.2)	669 (94.2)		
Unmarried/divorce	41 (5.8)	41 (5.8)		
Education, No. (%)			0	1
Middle school or less	48 (6.8)	48 (6.8)		
High school	98 (13.8)	98 (13.8)		
Technical secondary school	285 (40.1)	285 (40.1)		
College	222 (31.3)	222 (31.3)		
Master or higher	57 (8)	57 (8)		
Gravidity, No. (%)			0	1
Primigravida	342 (48.2)	342 (48.2)		
Multigravida	368 (51.8)	368 (51.8)		
Occupation, No. (%)			0	1
Fixed/self employed	658 (92.7)	658 (92.7)		
Not employed	52 (7.3)	52 (7.3)		
Gestational weeks, No. (%)			0	1
First trimester (<14)	489 (68.9)	489 (68.9)		
Second trimester (14–28)	193 (27.2)	193 (27.2)		
Third trimester (>28)	28 (3.9)	28 (3.9)		

*p*-values were calculated using the chi-square test.

*Statistically significant: *p* < 0.05.

**Table 4:** Results of PHQ-9, GAD, and PHQ-15 between the two matched groups.

Characteristics	COVID-19 epidemic (*n* = 710)	Pre-COVID-19 (*n* = 710)	*χ* ^2^	Value of *p*
PHQ-9 level, No. (%)			28.764	<0.001*
No depression symptoms	443 (62.4)	524 (73.8)		
Mild depression	199 (28)	158 (22.3)		
Moderate depression	54 (7.6)	20 (2.8)		
Moderate–severe depression	12 (1.7)	7 (1)		
Severe depression	2 (0.3)	1 (0.1)		
GAD-7 level, No. (%)			77.392	<0.001*
No anxiety symptoms	602 (84.8)	695 (97.9)		
Mild anxiety	92 (13)	13 (1.8)		
Moderate anxiety	13 (1.8)	1 (0.1)		
Severe anxiety	4 (0.4)	1 (0.1)		
PHQ-15 level, No. (%)			55.928	<0.001*
No somatic symptoms	282 (39.7)	408 (57.5)		
Mild somatic symptoms	280 (39.4)	231 (32.5)		
Moderate somatic symptoms	125 (17.5)	64 (9)		
Severe somatic symptoms	23 (3.2)	7 (1)		
Depression positive			17.875	<0.001*
PHQ-9 ≥ 10	68 (9.6)	28 (3.9)		
PHQ-9 < 10	642 (90.4)	682 (96.1)		
GAD positive			11.029	0.001
GAD-7 ≥ 10	16 (2.3)	2 (0.3)		
GAD-7 < 10	694 (97.7)	708 (99.7)		
Somatoform disorders positive			32.01	<0.001*
PHQ-15 ≥ 10	148 (20.8)	71 (10.0)		
PHQ-15 < 10	562 (79.2)	639 (90)		

*p*-values were calculated using the chi-square test. PHQ, Patient Health Questionnaire-15; GAD-7, Generalized Anxiety Disorder.

* Statistically significant: *p* < 0.05.

## Discussion

We investigated the impact of the COVID-19 pandemic on maternal mental health. We found that 9.5, 2.2, and 20.8% of pregnant women at their first antenatal care visit during the COVID-19 pandemic suffered from moderate-to-severe depression, anxiety, and somatoform symptoms, respectively. The prevalence of depression and anxiety in our study was significantly lower than that reported by other groups. For instance, a multicenter network study reported that 32% of Chinese pregnant women had depression (PHQ-9 ≥ 10) or anxiety (GAD-7 ≥ 5) during the COVID-19 pandemic (39). Jones et al. reported that anxiety and depression might have affected more than half of perinatal women during the first national lockdown in England (40). A multi-country network cross-sectional study in Europe reported that 15 and 11% of pregnant women had moderate-to-severe depressive symptoms (Edinburgh Depression Scale ≥ 13) and generalized anxiety symptoms (GAD ≥ 10) (26). However, the prevalence of depression in our study is lower than the 5.3% reported by Zhou et al. (21), comparable to the 9.8% reported by Wu et al. (41). These differences in prevalence might be due to different regions, study population, screening tools, and inconsistent cutoff points used to assess depression and anxiety. Overall, anxiety and depression are common mental challenges for pregnant women. Therefore, timely screening for mental health disorders and providing interventions is important for the health of pregnant women and their fetuses (42).

We found that 16.4, 4.4, and 25.7% of pregnant women had moderate-to-severe depression, anxiety, and somatoform symptoms during their first prenatal care before the COVID-19 attack, respectively, significantly higher than that during the COVID-19 pandemic. This finding was contrary to the results of previous studies and our expectations that pregnant women might have experienced higher risks of depression, anxiety, and distress under the stress of the COVID-19 outbreak (20, 30–32). We postulated that this outcome was partly due to better family support and less work pressure, which are protective factors for anxiety and depression among pregnant women. Nausea and vomiting are very common complaints during early pregnancy, affecting almost 60–80% of pregnant women (43). In addition, spontaneous abortion is the most common complication in the first trimester of pregnancy. These factors might be responsible for the high rates of anxiety and depression in early pregnancy. During the COVID-19 pandemic, Chongqing was under a comprehensive “lockdown,” including mandatory homestays, travel bans, and traffic controls (44). Staying at home kept pregnant women away from the pressure of work and gave them more time to recover from morning sickness. Moreover, more family companionship, support, and communication due to mandatory homestays may have reduced anxiety among pregnant women with regard to early pregnancy reactions, miscarriage, and mother role transition (45).

We also postulated that pregnant women with high levels of anxiety and depression are more likely to delay their expected first antenatal care or choose nearer health facilities for antenatal care due to their greater fear of contracting COVID-19 (46), which may partly explain the low prevalence of depression and anxiety among pregnant women in clinics during the COVID-19 pandemic. Our hospital is one of the comprehensive teaching hospitals with the largest scale and strongest professional skills in China. The obstetrics department is the diagnosis and treatment center for high-risk pregnancy in Chongqing and a prenatal diagnosis and fetal medicine center in western China. Before the COVID-19 outbreak, pregnant women with complicated comorbidities and complications were referred to our obstetrics department for antenatal care and delivery. High-risk pregnancy and pregnancy comorbidities or complications are independent risk factors for anxiety and depression in pregnant women (41, 47, 48). The lockdown may have prevented pregnant women with high-risk pregnancies outside the main urban areas of Chongqing from visiting our hospital for antenatal care during the COVID-19 pandemic, which might have led to the low prevalence of anxiety and depression in the obstetric clinic.

The contrasting outcomes were also attributed to mismatched distributions of demographic characteristics. There was a significantly low proportion of elderly women (>40 years), unemployed, low education level, ethnic minorities, multigravida, unmarried, or divorced cases in the first trimester among pregnant women during the COVID-19 pandemic than that in the pre-COVID-19 period. These demographic characteristics are closely related to anxiety and depression and are even considered to be risk factors for mental health disorders (2). Acheanpong et al. reported that elder maternal age and low educational level were significantly high among women with antenatal depression (49). In a previous meta-analysis, higher levels of education and better living conditions were found to be protective factors while low socioeconomic status was among the major risk factors (50). Ho-Fung et al. documented that unemployment is an associated risk factor for poor perinatal mental health (51). Wu et al. found that unmarried/divorced/widowed and unemployed cases in their first trimester of pregnancy had increased risks of antenatal anxiety and depression in the post-COVID-19 pandemic era (41). Multigravida and ethnic minorities also increase the risks of depression and anxiety among pregnant women (50, 52). Therefore, lower proportions of pregnant women with these potential risk factorsmight partly explain fewer pregnant women reported the symptoms of depression, anxiety, and somatoform disorders during the COVID-19 pandemic.

However, the significant differences in the distribution of socio-demographic characteristics between the two periods may not reflect the impact of the COVID-19 pandemic on the mental health of pregnant women. Propensity score matching is one of the statistical methods for controlling differences in variables between groups (53). After matching all sociodemographic characteristics with significant differences between the groups, we found that more pregnant women exhibited symptoms of depression, GAD, and somatoform disorders during the COVID-19 pandemic (2.3, 9.6, and 20.8%), relative to the pre-COVID-19 period (0.3, 3.9, and 10%). The results indicate that the COVID-19 pandemic increased the risks of anxiety and depression among pregnant women, consistent with evidence from previous studies. As a new and unknown viral disease, the COVID-19 pandemic may have caused stress, fear of illness, worries of infection, and unemployment, which in turn increased the risk of anxiety and depression (54, 55). Kakaraparthi et al. and Ayaz et al. found that the prevalence of anxiety and depression among pregnant women was significantly increased after the COVID-19 outbreak (pre-and post-COVID-19 outbreak) (30, 32). Zhou et al. and Berthelot et al. compared differences in the prevalence of mental health disorders between the different groups enrolled in the pre-and post-COVID-19 outbreak and reported consistent results (20, 31). Furthermore, it has been reported that up to 40–60% of pregnant women may have had anxiety and depression during the COVID-19 pandemic (19, 56). Therefore, we considered that pregnant women had higher risks of anxiety and depression during the COVID-19 pandemic. Unfortunately, a great proportion of pregnant women with anxiety and depression may not have attended the clinic for antenatal care. This phenomenon led to significantly low rates of anxiety and depression among pregnant women who came to clinics for their first antenatal care compared to the pre-pandemic period.

To our knowledge, our hospital is the only medical institution in Chongqing providing screening services for mental health disorders among pregnant women at their first antenatal care visit. In general, obstetricians might often pay more attention to the physical conditions of pregnant women and fetuses and overlook the mental health challenges. Currently, most medical institutions lack screening systems and corresponding intervention programs for maternal mental health disorders. We speculate that pregnant women with or at high risk for anxiety and depression may delay their prenatal care or change the healthcare facilities after public health emergencies indicated the necessity of developing a balanced healthcare system and accelerating the development of appropriate screening and intervention systems for maternal anxiety and depression in medical institutions at all levels. There is a need to understand the mental health of pregnant women who do not attend antenatal care after public health emergencies. Strengthening the publicity of mental health knowledge, improving the public’s ability to identify common psychological disorders such as anxiety and depression, and providing a network, convenient psychological evaluation systems, and decompression skills might be conducive to further promoting the mental health outcomes of pregnant women.

This study has some limitations. First, even though almost all pregnant women at their first antenatal care in our hospital during the COVID-19 pandemic were enrolled in our study, the sample size was small. Second, this study was only a single-center cross-sectional study, not a multicenter longitudinal follow-up study, which might have increased bias. Third, this study used the self-rating questionnaire to evaluate antenatal anxiety and depression among pregnant women, which might also have led to assessment bias. Hence, in future, a multicenter longitudinal follow-up study with a large sample size would be conducted to investigate the mental health disorders among pregnant women at different stages after the outbreak of public health emergencies by combining self-assessment and clinician-administered assessment scales.

## Conclusion

The COVID-19 pandemic increased the risk of mental health disorders among pregnant women. There were significant differences in the prevalence of anxiety, depression, and somatoform symptoms between the two COVID-19 periods before and after propensity score matching, which clarifies the impact of the COVID-19 pandemic on mental health among pregnant women and dispels the false impression that the prevalence of anxiety and depression among pregnant women at their first antenatal care in the clinic during the pandemic was significantly low. Our findings provide the theoretical evidence for promoting the balanced development of the medical system and the establishment of screening and intervention systems for maternal mental health disorders.

## Statement

We confirm that we have complied with our institution’s intellectual property regulations and there are no impediments to publication, including the timing of publication, with respect to intellectual property.

## Data availability statement

The raw data supporting the conclusions of this article will be made available by the authors, without undue reservation.

## Ethics statement

The studies involving human participants were reviewed and approved by ethics committee of the First Affiliated Hospital of Chongqing Medical University. The patients/participants provided their written informed consent to participate in this study.

## Author contributions

JG and XL analyzed the data and wrote the manuscript. JH, YG, AZ, and WC helped to design the questionnaire and collected the data. MA and QZ developed the online assessment systems. LC, SL, and XY helped the patients finish the questionnaires. LK conceived the experiments, was a fund provider, and was involved in writing the review. All authors contributed to the article and approved the submitted version.

## Funding

This study was supported by the medical research project of the Chongqing Health and Family Planning Commission (2018QNXM014) and the Chongqing Science and Health Joint Medical Scientific Research Project (2020MSXM079).

## Conflict of interest

The authors declare that the research was conducted in the absence of any commercial or financial relationships that could be construed as a potential conflict of interest.

## Publisher’s note

All claims expressed in this article are solely those of the authors and do not necessarily represent those of their affiliated organizations, or those of the publisher, the editors and the reviewers. Any product that may be evaluated in this article, or claim that may be made by its manufacturer, is not guaranteed or endorsed by the publisher.
